# Hometown attachment and corporate social responsibility: Evidence from overseas Chinese entrepreneurs

**DOI:** 10.3389/fpsyg.2022.943701

**Published:** 2022-09-23

**Authors:** Jiahui Xia, Zhanchi Wu, Zhaolan Dang, Rui Zhang

**Affiliations:** ^1^School of Management, Jinan University, Guangzhou, China; ^2^Department of Economics, Jinan University, Guangzhou, China

**Keywords:** hometown attachment (HA), corporate social responsibility (CSR), migrants, overseas Chinese, social trust

## Abstract

The relationship between hometown attachment (HA) and corporate social responsibility (CSR) is a topic to be explored in depth. We measured the HA by the Chinese diaspora background and the immigrant culture of the ultimate controllers of the firm and employed the sample of Chinese non-financial private-listed companies in Shanghai and Shenzhen from 2003 to 2019 to investigate the impact of the HA on overseas Chinese entrepreneurs on CSR. We found that the HA of the overseas Chinese ultimate controller significantly increases the level of CSR, and this promoting effect rises when the firm ultimately held by the overseas Chinese entrepreneur is registered in the expatriate hometown. Our further analysis found that the personal characteristics of overseas Chinese entrepreneurs and regional cultural differences have a moderating effect on the above relationship. In particular, we found that overseas Chinese entrepreneurs who are women or with lower academic qualifications have a stronger sense of CSR. Moreover, in areas with low marketization or a high level of social trust, HA of overseas Chinese entrepreneurs plays a more active role in CSR. The results remain robust after the robustness test and the endogenous test. The conclusion of this study not only highlights the impact of psychological factors on the level of CSR but also provides a reference for the study of the decision-making behavior of overseas Chinese entrepreneurs.

## Introduction

Corporate social responsibility (CSR) is in the spotlight as a corporate strategy for sustainable development and competitiveness of companies (Crane and Matten, 2020). Existing literature has explored the drivers of CSR from the perspectives of agency issues, corporate reputation, investors, etc., Masulis and Reza (2015), Tetrault Sirsly and Lvina (2019), and Chen et al. (2020). In addition, there are articles exploring CSR from environmental perspectives such as eco-innovation, for example, Zaušková and Rezníčková (2020) and Burke and Zvarikova (2021). Whether and how the characteristics of corporate managers, especially psychological factors, affect CSR is still a hot issue worth exploring. The literature has explored the impact of psychological factors of corporate executives on CSR from the perspectives of personality and family (Petrenko et al., 2016; Cronqvist and Yu, 2017; Davidson et al., 2019). However, with increasing globalization, the scale of migration has gradually expanded, but the impact of the psychology of migration on CSR remains less explored.

Hometown attachment (HA) is a prevalent psychological factor among immigrants (Huang et al., 2018). Specifically, not adapting to a foreign culture or missing family members makes immigrants keep in touch with people in their hometown, such as returning to their hometown to start a business and remittances (Harris, 2015; Kong et al., 2019). Furthermore, existing literature studied the impact of hometown ties on firms, such as innovation (Ren et al., 2021a,b), trade credit (Kong et al., 2020), tax avoidance (Shen et al., 2019), and stock investment (Coval and Moskowitz, 1999). Although Zhang et al. (2018) studied the impact of directors with overseas experience on CSR, the main consideration was the professional and academic experience of executives. Especially, the existing literature failed to explore the impact of hometown connection on CSR from a psychological perspective. Therefore, we attempted to enrich the literature in this area and explored the impact of HA on CSR based on overseas migration. Considering the possible agency conflicts between shareholders and executives, we used the ultimate controller of the company as the research object.

Using the ordinary least squares (OLS) regression analysis, we developed two linear regression models to examine the effects and mechanisms of HA on CSR. First, we empirically examined the relationship between HA and CSR among ultimate controllers who emigrated overseas, using a sample of Chinese non-financial private-listed companies from 2003 to 2019. Furthermore, we implemented heterogeneity tests for the ultimate controller characteristics (i.e., gender, age, and education) and regional characteristics (i.e., marketization and social trust).

Finally, to address possible endogeneity issues, we used the 2SLS instrumental variable method for endogeneity testing. Specifically, we selected two instrumental variables. One is the number of coastal ports of commerce that China was forced to open between 1840 and the end of the 19th century under unequal treaties as well as under *The Tongzhi Charter*. The other is a dummy variable when Christian and Roman Catholic missionaries opened Western-style universities throughout China as of the 1820s. We documented that the greater the number of open coastal ports of commerce and the opening of Western-style universities were more likely to increase the number of locals emigrating overseas. In addition, we performed robustness tests using proxy variables for key variables and excluding the effect of the economic crisis.

The contributions of our study to the literature are mainly in three aspects. First, this study enriched the study of CSR drivers. We extended the psychological factor HA to CSR research and explored new drivers of CSR from the perspective of the ultimate controller who migrates overseas. In this study, we further examined the influence of the personal traits of the ultimate controller and the external environment on CSR. Second, we further expanded on the economic consequences of attachment psychology. Some research studies focused on the impact of hometown ties on corporate managers. However, in exploring how early migration abroad shapes individual character, this study contributes to a more comprehensive understanding of how attachment psychology from managers' migration experiences affects firm strategy. Third, we promoted interdisciplinary research by combining psychology with business management. Specially, we complemented the impact of psychological factors brought about by the immigrant experience on business practices.

The rest of the paper is organized as follows. Section Literature review and hypothesis development discusses the relevant literature and presents our hypotheses. Section Data, variables, and methodology describes the sample, the main variables, the research design, and the descriptive statistics. Section Empirical results and analysis presents the empirical results and analysis. Section Robustness tests presents the endogeneity test and the robustness test. Section Conclusions and discussions presents the conclusion and discussion.

## Literature review and hypothesis development

Overseas immigrants have an attachment to their home countries (Wessendorf, 2010; Huang et al., 2018; Palladino, 2019; Wang et al., 2020; Zou et al., 2021). Due to attachment psychology, migrants actively seek ties with their home countries. On the one hand, immigrants retain the culture and identity of their home country after they arrive in the host country (Berry et al., 2006). Meanwhile, immigrants come together to join local hometown communities and maintain hometown food traditions (Ma and Xiang, 1998). For example, Chinatown is one of the iconic places where overseas Chinese meet and where they make friends, find jobs, and participate in traditional cultural activities (Zhou, 2009). On the other hand, immigrants establish ties with their home countries, such as returning to their home countries to start businesses and contact their families. Specifically, King and Christou (2014) found that second-generation Greek-Germans and Greek-Americans return to Greece out of a strong Greek identity and to reconstruct their ties to their home country. Therefore, expatriates identify with a shared culture and identity, which implicitly influences individual decisions and behaviors in life and work (Anton and Lawrence, 2014).

Attachment drives expatriates to do good things for their hometown. In detail, when leaving their hometown to live and work in another place, individuals favor the people and things of their hometown. For example, Hodler and Raschky (2014) surveyed national leaders in 126 countries and found that regional favoritism existed during the time these leaders were in power with stronger nighttime lighting in their birthplaces. In addition, when their executives had hometown ties to the firms, firms were more innovation-oriented (Ren et al., 2021a), and the availability of trade credit to firms increased (Kong et al., 2020). At the same time, attachment promotes environmental responsibility (Lee, 2011), concern for climate change (Scannell and Gifford, 2013), and the implementation of sustainable development goals (Adger et al., 2013; Gifford, 2014; Masterson et al., 2017; Brown et al., 2019).

According to the upper echelons theory, company activities are influenced by managerial background characteristics (Hambrick and Mason, 1984; Michel and Hambrick, 1992; Aktas et al., 2016). In particular, the ultimate controller of the company has the power to make decisions about the company and has a significant influence on the development of the corporate strategy (Watanabe, 2002). Notably, CSR is an important decision for companies, having a significant positive impact on both organizational performance and competitiveness (Ahmed and Streimikiene, 2021). Therefore, the characteristics of the company's ultimate controller have an impact on CSR decisions.

Thus, returning to their home countries to invest and start businesses after leaving their home countries reflects the HA of the ultimate controllers who emigrate overseas. Additionally, HA encompasses identification with traditional culture, dependence on social networks based on blood and local ties, and a sense of responsibility for building the home country. In summary, we proposed the following hypothesis:

H1: Hometown attachment of ultimate controllers who emigrate abroad is positively associated with CSR.

In China, the hometowns of overseas Chinese are distributed all over the country but are mainly concentrated in five provinces – Guangdong, Fujian, Guangxi, Hainan, and Zhejiang – called expatriate hometowns. There are two basic prerequisites for the formation of an expatriate hometown. One is that the population and descendants of immigrants from the region who have emigrated overseas have reached a certain size. The other is that these overseas immigrants and their descendants maintain closer ties with the region (Dai and Song, 1998). Specifically, the main characteristics of overseas Chinese hometowns include a large number of overseas Chinese families, close ties at home and abroad, many overseas remittances, rich overseas social resources, and a diverse Chinese and Western culture (Wang, 2001). Thus, overseas Chinese have strong ties with their hometowns, both in historical times and in contemporary times (Zheng, 2009).

Expatriate hometown has both a strong traditional Chinese culture and a diverse overseas immigrant culture. Allen et al. (2005) found that China's economic growth relies more on informal institutions than on formal ones. As the ultimate controller and decision-maker, overseas Chinese entrepreneurs are influenced by their cultural environment despite their personal behavioral preferences (Zingales, 2015). Being in an expatriate hometown, overseas Chinese entrepreneurs unconsciously put themselves in the role of “hometown people” and make business decisions not only considering corporate profits but also taking into account the interests of stakeholders in their hometown. Moreover, the local people in the expatriate hometown have frequent and active contact with overseas Chinese. Customer transactions, government-enterprise exchanges, and community exchanges convey the traditional Chinese culture's ethical responsibility of “return to one's hometown in silken robes” and “honoring one's ancestors.” Influenced by the strong traditional ideas of overseas Chinese hometowns, overseas Chinese consciously improve the level of CSR. Therefore, we further proposed the second hypothesis:

H2: When the place of incorporation is located in the expatriate hometown, the hometown attachment of the ultimate controller who immigrated overseas is positively related to CSR.

As a decision-maker, the personality, values, and upbringing of the ultimate controller potentially affect business operations and corporate culture (Norburn and Birley, 1988; Wiersema and Bantel, 1992). For example, Crisan-Mitra et al. (2020) identified six patterns of management behavior in implementing CSR and found that managers' personal traits differentially affect corporate social performance. Furthermore, Dubno (1985) found that women are gentler and more sensitive compared to men. As a result, female ultimate controllers who emigrate overseas may have a deeper attachment to their hometowns and, in turn, be more willing to do good for them and increase their level of CSR. In addition, as immigrants grow older, their attachment to their home countries may gradually deepen. As ultimate controllers, expatriates may be more inclined to increase their level of CSR to express attachment. Finally, Hu et al. (2021) found that studying abroad helped develop an individual's dialectical thinking. Entrepreneurs, who emigrate abroad embrace the diversity of thought, may make more rational decisions and make fewer decisions due to attachment when running their businesses. Thus, we formulated the following hypotheses:

H3a: Female ultimate controllers positively moderate the relationship between hometown attachment and CSR.
H3b: The age of the ultimate controller positively moderates the relationship between hometown attachment and CSR.
H3c: Ultimate controller's education level negatively moderates the relationship between hometown attachment and CSR.

The market environment has a key influence on business decisions (Narver and Slater, 1990). In regions with low marketization, product markets, production factors, and legal systems are relatively imperfect. The ultimate controllers of enterprises are subject to weaker supervision and constraints, and their behavioral decisions are more influenced by traditional culture. In contrast, in regions with high marketization, more efficient total factor productivity and better legal systems may prompt ultimate controllers to focus more on corporate profits, crowding out attention to CSR. Therefore, this study proposes the fourth hypothesis as follows:

H4: The degree of marketization negatively moderates the relationship between hometown attachment and CSR.

As an informal institution, social trust guides business decision-makers to comply with the rules of the trade and reminds them of the high costs associated with the breach of trust (Trevino, 1986; Chen et al., 2004). In China, people value relationships, and the ultimate controller who has emigrated overseas needs to gain the trust of the locals to start a business and invest in their home country. Additionally, reciprocity is one of the most important ways to gain trust (Berg et al., 1995). By voluntarily giving up a portion of profits to fulfill their social responsibilities, firms build mutual trust with locals to maintain complex social networks. Moreover, May et al. (2021) found that organizational trust positively moderates the relationship between employee green behavior and corporate environmental responsibility. Therefore, we inferred that in regions with high levels of social trust, the HA of the ultimate controllers who migrate overseas is more conducive to CSR. As a result, we proposed the following hypothesis:

H5: Social trust positively moderates the relationship between hometown attachment and CSR.

## Data, variables, and methodology

### Data

Our sample includes private firms listed on both the Shanghai and Shenzhen securities exchanges in the period from 2003 to 2019. First, we downloaded the annual reports of listed companies from the website of *cninf* ^1^ Since the background information of the ultimate controller has been disclosed in the annual reports since 2003, we used 2003 as the starting point for our data search. Then, we manually searched for information on the company's ultimate controller from the “Share Changes and Shareholders” section of the company's annual report. In addition, we studied companies whose ultimate controller is an overseas Chinese, and correspondingly, our control group included companies whose ultimate controller is an individual and not an overseas Chinese, thus we excluded the sample where the ultimate controller is a legal entity. Furthermore, when a company discloses multiple ultimate controllers and the difference in individual shareholdings exceeds 10%, we determined that the higher shareholding is the ultimate controller. Moreover, if the difference in individual shareholdings is within 10%, we determined that the person who held the highest position within the company is the ultimate controller. Then, we excluded the sample of companies in the financial sector because financial institutions such as banks and insurance have significant operational differences from other industries. In addition, we excluded the sample of companies with special treatment (ST^*^ or ST) because they had significant operational problems. Finally, to ensure the continuity and comparability of sample information, we excluded the sample of companies with less than 3 years of data. Thus far, the effective sample size is 12,385, including 1,465 listed firms whose ultimate controller is an individual. To reduce the effect of outliers, we carried out winsorization of our sample at 1% on the continuous variable in each tail.

Moreover, we manually supplemented key information on nationality and permanent residency abroad (including a residency in Hong Kong and Macau) through the websites of National and provincial federations of returned overseas Chinese^2^. Additionally, we supplemented the information of the ultimate controller from sites such as Baidu and Google. Moreover, we obtained financial data from the China Stock Market & Accounting Research (CSMAR) database. Table 1 displays the industry composition. Additionally, Table 2 shows the percentage of overseas Chinese ultimate controllers of the observed sample companies, and Table 3 presents descriptive statistics including company financial data and ultimate controller information^3^.

**Table 1:** Industry distribution of sample firms.

**Industry**	**Number of firms**	**Percentage**
Agriculture, forestry, animal husbandry, and fishery	24	1.64%
Mining	15	1.02%
Manufacturing	1,089	74.33%
Electricity, heat, gas and water production, and supply industry	6	0.41%
Construction	27	1.84%
Wholesale and retail	46	3.14%
Transportation, storage, and postal industry	12	0.82%
Accommodation and catering industry	2	0.14%
Information transmission, software, and information technology services	122	8.33%
Real estate	47	3.21%
Leasing and business services	12	0.82%
Scientific research and technical services	13	0.89%
Water, environment, and public facilities management industry	6	0.41%
Residential services, repairs, and other services	5	0.34%
Health and social work	2	0.14%
Culture, sports, and entertainment industry	11	0.75%
Comprehensive industry	26	1.77%

The industry classification is based on the Tier 1 classification of *the Industry Classification Guidelines for Listed Companies (Revised in 2012)* issued by the China Securities Regulatory Commission, with a total of 19 industries. Additionally, we excluded the financial industry. Moreover, the sample of companies in the education industry is 0. Therefore, Table 1 illustrates the sample distribution of 17 industries.

**Table 2:** Year distribution of ultimate controllers.

**Year**	**Overseas Chinese**		**Non-overseas Chinese**		**Total**
	**Number**	**Percentage**	**Number**	**Percentage**	
2003	8	14.55%	47	85.45%	55
2004	16	9.64%	150	90.36%	166
2005	25	11.31%	196	88.69%	221
2006	25	10.29%	218	89.71%	243
2007	25	9.26%	245	90.74%	270
2008	39	10.68%	326	89.32%	365
2009	45	10.77%	373	89.23%	418
2010	61	11.66%	462	88.34%	523
2011	95	11.11%	760	88.89%	855
2012	121	11.20%	959	88.80%	1,080
2013	124	10.38%	1,071	89.62%	1,195
2014	128	10.41%	1,101	89.59%	1,229
2015	137	10.33%	1,189	89.67%	1,326
2016	140	10.36%	1,212	89.64%	1,352
2017	136	10.21%	1,196	89.79%	1,332
2018	132	10.38%	1,140	89.62%	1,272
2019	50	10.35%	433	89.65%	483

**Table 3:** Descriptive statistics.

**Variables**	**Obs**	**Mean**	**Std.Dev**.	**Min**	**Median**	**Max**
*CSR_rate*	12385	0.1209	0.0668	0.0177	0.1061	0.3880
*HA_hqhr*	12385	0.1055	0.3072	0.0000	0.0000	1.0000
*HA_qx*	12385	0.0571	0.2320	0.0000	0.0000	1.0000
Firm characteristics						
*Size*	12385	21.7081	1.0547	19.2928	21.6173	24.8217
*Leverage*	12385	0.3903	0.1975	0.0467	0.3835	0.8783
*Growth*	12385	0.2453	0.5857	−0.6204	0.1478	4.7108
*Cash*	12385	0.0404	0.0740	−0.2038	0.0403	0.2490
*Roa*	12385	0.0409	0.0586	−0.2446	0.0402	0.2051
*Firmage*	12385	7.4535	5.7094	0.0000	6.0000	23.0000
*Roe*	12385	0.0610	0.1297	−0.8514	0.0684	0.3491
*Manho*	12385	0.1947	0.2141	0.0000	0.1019	0.7059
*Mtnum*	12385	3.2717	0.6995	3.0000	3.0000	6.0000
*Ucpd*	12385	0.8107	0.3918	0.0000	1.0000	1.0000
*Wedge*	12385	0.5460	0.4979	0.0000	1.0000	1.0000
Ultimate controller's characteristics						
*Ucsgender*	12385	0.9523	0.2132	0.0000	1.0000	1.0000
*Ucsage*	12385	0.5284	0.0805	0.3400	0.5200	0.7400
*Ucsedu*	12385	3.3908	1.2403	1.0000	3.0000	6.0000
Regional characteristics						
*GDP*	12385	64664.6875	29985.2578	9440.0000	63374.0000	153095.0000
*Religion*	12385	10.4442	3.7476	5.0000	11.0000	15.0000
*Chamber*	12385	41.8470	29.7919	1.0000	37.0000	103.0000

### Variables

#### Hometown attachment

Following Hu et al. (2017), we employed the identity of the ultimate controller and the firm's registration site to measure HA. In detail, we created two dummy variables to test H1 and H2, respectively. The first dummy variable is *HA_hqhr*, defined to be equal to 1 when the ultimate controller of the firm is overseas Chinese and 0 otherwise.

Considering regional culture, we set the second dummy variable as *HA_qx*, which is defined to be equal to 1 when the registered place of the firms with overseas Chinese ultimate controller is located in expatriate hometown, that is, Guangdong, Fujian, Guangxi, Hainan, and Zhejiang, and 0 otherwise.

#### Corporate social responsibility

Following Song and Li (2010), we used the corporate social contribution rate, *CSR_rate*, to measure CSR. In detail, *CSR_rate* is calculated as (Taxes paid - tax refunds received + cash paid for dividends, profits or interest payments + cash paid to and for employees + donations + sponsorship expenses - disposal of pollution and cleanup costs)/average total assets. This indicator evaluates the fulfillment of social responsibility to stakeholders such as the government, employees, investors, and communities using the company's financial data. The higher the *CSR_rate* the better the CSR performance.

#### Control variables

We controlled for a number of factors that could potentially affect CSR, including firm level, individual level, and regional level.

##### Firm-level control variables

Referring to Chen et al. (2020) and Ren et al. (2021a), we introduced control variables at the firm level, including the following:

Firm size (*Size*). We used the natural logarithm of total assets to measure firm size.

Financial leverage (*Leverage*). We employed the total debt divided by total assets to measure financial leverage.

Growth (*Growth*). We introduced the ratio of operating revenue growth to measure a firm's growth.

Operating cash flows (*Cash*). We used the net of operating cash flows divided by total assets to measure the operating cash flows of the firm.

Profitability (*Roa*). We used return on assets to measure the firm's profitability.

Years of listing (*Firmage*). We used the current year minus the IPO year to measure the firm's years of listing.

Firm performance (*Roe*). We introduced return on equity to measure firm performance.

Management shareholding (*Manho*). We employed the number of shares held by management scaled by the total number of shares to measure management shareholding.

Size of the board of supervisors (*Mtnum*). We used the number of supervisors to measure the size of the board of supervisors.

Whether the ultimate controller is the chairperson or general manager (*Ucpd*). We introduced a dummy variable to measure whether the ultimate controller is the chairman or general manager and it equals 1 if the ultimate controller concurrently serves as the chairperson or CEO, and 0 otherwise.

Separation of powers (*Wedge*). We employed a dummy variable to measure the separation of powers and it equals 1 if the control right and ownership of the ultimate controller are separated, and 0 otherwise.

##### Ultimate controller characteristic variables

In addition, following Kong et al. (2020), we controlled the ultimate controller characteristic variables, including the following:

Ultimate controller's gender (*Ucsgender*). We employed a dummy variable to measure the ultimate controller's gender and if the ultimate controller is male it equals 1, and 0 otherwise.

Ultimate controller's age (*Ucsage*). We introduced the age of the ultimate controller divided by 100 to measure the ultimate controller's age.

Ultimate controller's education (*Ucsedu*). We used a continuous numerical proxy for the education level of the ultimate controller. Specifically, 1 = technical secondary school or below, 2 = junior college, 3 = undergraduate, 4 = master, 5 = doctor, and 6 = other.

##### Regional characteristics variables

Finally, we followed Ren et al. (2021a) and controlled the regional variables, including the following:

GDP (*GDP*). GDP divided by the total population of the province where the firm is located is employed for this measurement (Data sources: the website of the National Bureau of Statistics website^4^.

Religion (*Religion*). This variable is measured by the number of award-winning religious places in the province where the firm is located (Data sources: the website of the National Religious Affairs Administration^5^).

Chamber (*Chamber*). We introduced the number of chambers of commerce in the province where the firm is located to measure this variable (Data sources: the website of the Federation of Industry and Commerce of each province^6^).

### Regression models

To examine the effect of the ultimate controller's HA on CSR, we used the following baseline regression model.


(1) $$\begin{array}{l} CSR\text{\_}rate\;=\;\alpha_{o}+\alpha_{1}HA\text{\_}hqhr+\alpha_{2}Controls\;+\;Year\\ \;+Industry+\varepsilon \end{array}$$

(2) $$\begin{array}{l} CSR\text{\_}rate\;=\;\alpha_{o}+\alpha_{1}HA\text{\_}qx+\alpha_{2}Controls\;+\;Year\\ \;+Industry+\varepsilon \end{array}$$

where the dependent variable (*CSR_rate*) is the level of CSR measured by the corporate social contribution rate. In model 1, the independent variable *HA_hqhr* is a dummy variable that equals 1 if the ultimate controller of a firm is overseas Chinese, and 0 otherwise. In model 2, the independent variable *HA_qx* is also a dummy variable that equals 1 if the registration site of the firm controlled by an overseas Chinese (i.e., *HA_hqhr*=1) is in an expatriate hometown, and 0 otherwise. *Controls* is a vector of control variables defined in Table 1. We also controlled the year- and industry-fixed effects in all models to control the unobservable factors. Model 1 and model 2 examine the H1 and H2, respectively. In all models, we expected that the coefficient α_1_ is significantly positive, that is, HA of the firms' ultimate controllers is significantly positively correlated with the level of CSR.

**Figure 1:**
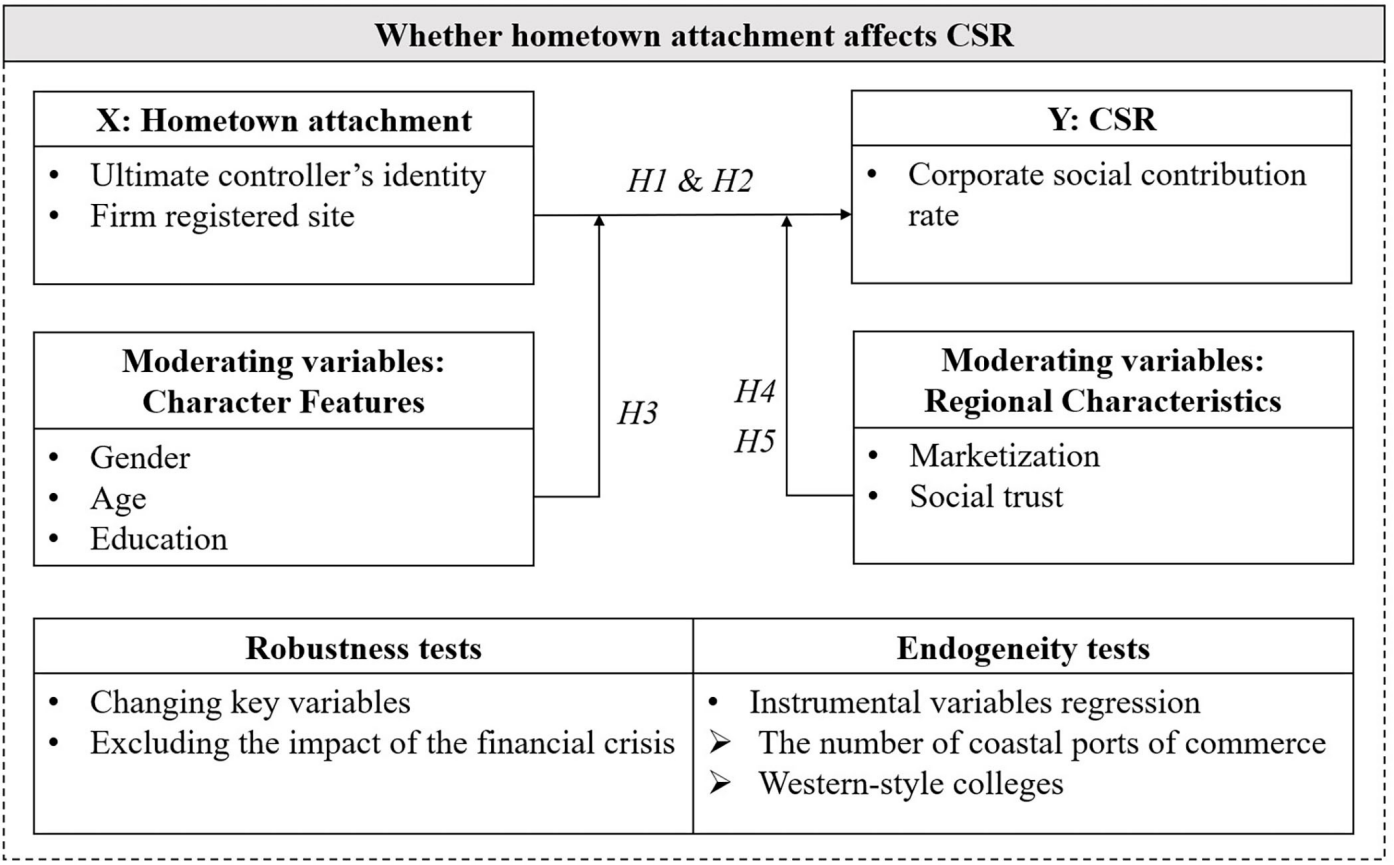
Analysis framework of research questions.

To sum up, Figure 1 illustrates the identification strategy. Specifically, we first tested whether HA affects CSR. We used two independent variables to proxy HA, one based on the overseas Chinese identity of the ultimate controller (*HA_hqhr*) and the other based on the location of the company's registration (*HA_qx*). Next, we implemented heterogeneity tests for the characteristics of the ultimate controller (i.e., gender, age, and education) and regional characteristics (i.e., marketization and social trust). In addition, to test the robustness of the results, we re-tested models 1 and 2 by replacing key variables and excluding the effect of the economic crisis. Finally, to exclude possible endogeneity issues, we used instrumental variables for endogeneity testing through 2SLS regressions.

### Descriptive statistics

Table 1 presents the distribution of companies by industry. It is shown that the manufacturing sector accounts for the largest share of the 1,465 companies in the sample, with 74.33%. Next, there are 122 companies in information transmission, software, and information technology services, accounting for 8.33%. In addition, there are 47 real estate companies, with 3.21%. Moreover, 46 companies are in wholesale and retail, accounting for 3.14%. Additionally, construction companies have 27 companies, with 1.84%. Also, there are 26 companies in the comprehensive industry, accounting for 1.77%. In addition, agriculture, forestry, animal husbandry, and fishery companies have 24 companies, accounting for 1.64%.

Table 2 exhibits the yearly distribution of companies whose ultimate controllers are individuals. It is shown that the number of companies whose ultimate controllers are overseas Chinese has increased almost yearly from 2003 to 2019, and the percentage, although fluctuating, has remained almost stable in the range from 10 to 11%. In addition, from our sample statistics, the total number of companies announcing in their annual reports that the ultimate controller is an individual increases almost year by year, indicating the increasingly important role of individual ultimate controllers.

Table 3 represents the descriptive statistics of the variables. *CSR_rate* ranges from 0.0177 to 0.3880, and the mean of *CSR_rate* is 0.1209. In all firm year samples, 10.55% of the ultimate controller are overseas Chinese and 5.71% of the firms held by overseas Chinese are registered in expatriate hometowns. In terms of the control variables, the average firm size (*Size*) is 21.7081, financial leverage (*Leverage*) is 39.03%, and ROA (*Roa*) is 4.09%. Additionally, the mean of the ratio of operating revenue growth (*Growth*) is 24.53%. Moreover, the mean of operating cash flows (*Cash*) is 4.04%. Furthermore, the average listing year (*Firmage*) is 7.4535 years. In addition, the average firm performance (*Roe*) is 6.10%. Then, for the management shareholding (*Manho*), the average is 19.47%. Moreover, the mean of the size of the board of supervisors (*Mtnum*) is 3.2717. In addition, there are 81.07% of the companies whose ultimate controller is the chairman or general manager (*Ucpd*). Furthermore, there are 54.60% of the companies where the control right and ownership of the ultimate controller are separated (*Wedge*). For ultimate controller's characteristics, it is shown that 95.23% of ultimate controllers are male (*Ucsgender*), and the average age of ultimate controllers is 52.84 years old (*Ucsage*). In addition, the average education of ultimate controllers is undergraduate (*Ucsedu*). For regional characteristics, the average GDP per capita level by province is 64664.6875RMB. In addition, the average number of award-winning religious places in the province is 10.4442, and the average of number of chambers of commerce in the province is 41.8470.

Table 4 reports the results of the univariate test. We compared the level of CSR (*CSR_rate*) and the ultimate controller's characteristics between firms with and without overseas Chinese ultimate controller (*HA_hqhr*), and between firms located or not located in expatriate hometown (*HA_qx*). The results show that the average CSR level of the firms with overseas Chinese ultimate controller is 0.1317, while firms with no overseas Chinese ultimate controller is 0.1230, which exists a statistically significant difference (0.87 percentage points) at the 1% level. Moreover, for the firms with overseas Chinese ultimate controller, the average CSR level of the firms registered in famous overseas Chinese hometown is 0.1354, whereas firms registered in other places is 0.1232; the difference (1.22 percentage points) is statistically significant at a 1% level.

**Table 4:** Univariate test.

	**N**	**Mean**	**N**	**Mean**	
	*HA_hqhr* = 0		*HA_hqhr* = 1		Difference
*CSR_rate*	11078	0.1230	1307	0.1317	−0.0087^***^
*Ucsgender*	11078	0.9548	1307	0.9290	0.0258^***^
*Ucsage*	11078	0.5285	1307	0.5274	0.0012
*Ucsedu*	11078	3.3882	1307	3.5293	−0.1411^***^
	*HA_qx* = 0		*HA_qx* = 1		Difference
*CSR_rate*	11678	0.1232	707	0.1354	−0.0122^***^
*Ucsgender*	11678	0.9537	707	0.9234	0.0304^***^
*Ucsage*	11678	0.5282	707	0.5317	−0.0035
*Ucsedu*	11678	3.3998	707	3.4635	−0.0637

*** *p* < 0.01.

## Empirical results and analysis

### The effect of ultimate controller's HA on CSR

We first examined whether the firm's ultimate controller is an overseas Chinese affecting CSR. Table 5 reports the baseline regression results of H1. Specifically, column 1 of Table 5 tests the relationship of the dependent variable *CSR_rate* and the independent variable *HA_hqhr*, and only controls the variables of firm-level characteristics and the year and industry dummies. It is observed that *HA_hqhr* has a positive correlation with *CSR_rate* with an estimated coefficient of 0.0066 and is significant at a 1% significance level. Then, we gradually added the characteristics of the ultimate controller and region. In detail, after adding the ultimate controller characteristic variables in column 2, *HA_hqhr* and *CSR_rate* are positively correlated with an estimated coefficient of 0.0068 and are significant at the 1% level of significance. Finally, after we added all control variables in column 3, the estimate coefficient of *HA_hqhr* has a positive estimate of 0.0062 with a *t*-statistics of 3.46 and the relationship is significant at the 1% level of significance. The above tests all control year and industry dummy variables. Therefore, the baseline results support H1, that is, HA of the ultimate controller promotes CSR.

**Table 5:** The effect of the ultimate controller's HA on CSR (H1).

**Variables**	**(1)**	**(2)**	**(3)**
	***CSR_rate***	***CSR_rate***	***CSR_rate***
*HA_hqhr*	0.0066*** (3.70)	0.0068*** (3.81)	0.0062*** (3.46)
*Size*	−0.0061*** (−8.87)	−0.0062*** (−8.90)	−0.0062*** (−9.00)
*Leverage*	0.0456*** (11.46)	0.0453*** (11.39)	0.0462*** (11.58)
*Growth*	−0.0019 (−1.60)	−0.0019 (−1.62)	−0.0019 (−1.63)
*Cash*	0.1644*** (18.85)	0.1639*** (18.73)	0.1644*** (18.73)
*Roa*	0.6461*** (20.75)	0.6477*** (20.77)	0.6479*** (20.78)
*Firmage*	−0.0007*** (−5.21)	−0.0006*** (−5.11)	−0.0007*** (−5.05)
*Roe*	−0.1238*** (−9.80)	−0.1244*** (−9.83)	−0.1243*** (−9.83)
*Manho*	0.0038 (1.03)	0.0030 (0.83)	0.0025 (0.69)
*Mtnum*	0.0031*** (4.21)	0.0031*** (4.25)	0.0035*** (4.69)
*Ucpd*	0.0016 (1.13)	0.0015 (1.08)	0.0014 (0.97)
*Wedge*	0.0058*** (4.35)	0.0056*** (4.23)	0.0059*** (4.44)
*Ucsgender*		0.0058** (2.42)	0.0061** (2.55)
*Ucsage*		−0.0119* (−1.74)	−0.0122* (−1.79)
*Ucsedu*		−0.0009** (−2.04)	−0.0010** (−2.32)
*GDP*			0.0000** (2.11)
*Religion*			−0.0005*** (−3.20)
*Chamber*			0.0000 (1.55)
*Constant*	0.1380*** (8.99)	0.1417*** (9.08)	0.1466*** (9.32)
*Industry*	YES	YES	YES
*Year*	YES	YES	YES
*N*	12,385	12,385	12,385
*Adj.R^2^*	0.268	0.269	0.270

Robust t-statistics in parentheses. ***p < 0.01, ** *p* < 0.05, * *p* < 0.1.

For control variables, the estimate coefficient of *Size* and *CSR_rate* has a negative estimate of −0.0062 and is significant in 1% level significance in column 3 of Table 5, consistent with Udayasankar (2008). Moreover, the coefficient of *Leverage* is 0.0462, which is significantly positive at the 1% level of significance in column 3. In addition, *Cash, Roa, Mtnum*, and *Wedge* have a significant positive relationship with *CSR_rate*, respectively. Moreover, *Firmage* and *Roe* have a significant negative relationship with *CSR_rate*. For the variables of ultimate controller trait, *Ucsgender, Ucsage*, and *Ucsedu* are significantly correlated with *CSR_rate*. For regional characteristic variables, *GDP* is positively correlated with *CSR_rate*, whereas *Religion* is negatively correlated with *CSR_rate*. Overall, H1 is supported, that is, the HA of the ultimate controller has a positive impact on CSR.

Next, we examined whether the registration site of the firm with an overseas Chinese ultimate controller in an expatriate hometown influences CSR. Table 6 reports the regression results of H2. In column 1, we controlled firm characteristics variables and year- and industry-fixed effects. The results showed that the estimate coefficient of *HA_qx* has a positive estimate of 0.0106 with a *t*-statistic of 4.30. Then, in column 2 of Table 6, we added the variable of ultimate controller characteristics. It is shown that *HA_qx* was positively correlated with *CSR_rate* with an estimated coefficient of 0.0110, which was significant at 1% significance level. Moreover, in column 3 of Table 6, we further added the regional characteristics variables and it is shown that *HA_qx* is positively correlated with *CSR_rate* with an estimated coefficient of 0.0108, remaining statistically significant at 1% significance level. Therefore, the results are robust.

**Table 6:** The effect of the ultimate controller's HA on CSR (H2).

**Variables**	**(1)**	**(2)**	**(3)**
	***CSR_rate***	***CSR_rate***	***CSR_rate***
*HA_qx*	0.0106*** (4.30)	0.0110*** (4.42)	0.0108*** (4.31)
*Size*	−0.0061*** (−8.84)	−0.0061*** (−8.86)	−0.0062*** (−8.96)
*Leverage*	0.0452*** (11.34)	0.0449*** (11.27)	0.0459*** (11.48)
*Growth*	−0.0018 (−1.56)	−0.0019 (−1.57)	−0.0019 (−1.59)
*Cash*	0.1642*** (18.85)	0.1638*** (18.73)	0.1645*** (18.77)
*Roa*	0.6454*** (20.68)	0.6471*** (20.69)	0.6471*** (20.71)
*Firmage*	−0.0007*** (−5.18)	−0.0006*** (−5.08)	−0.0007*** (−5.04)
*Roe*	−0.1234*** (−9.74)	−0.1241*** (−9.77)	−0.1239*** (−9.76)
*Manho*	0.0036 (1.00)	0.0029 (0.79)	0.0025 (0.68)
*Mtnum*	0.0032*** (4.27)	0.0032*** (4.32)	0.0036*** (4.79)
*Ucpd*	0.0013 (0.97)	0.0013 (0.92)	0.0011 (0.82)
*Wedge*	0.0057*** (4.33)	0.0056*** (4.20)	0.0059*** (4.42)
*Ucsgender*		0.0060** (2.50)	0.0064*** (2.64)
*Ucsage*		−0.0124* (−1.81)	−0.0128* (−1.87)
*Ucsedu*		−0.0008** (−1.98)	−0.0010** (−2.30)
*GDP*			0.0000** (2.35)
*Religion*			−0.0005*** (−3.23)
*Chamber*			0.0000 (1.06)
*Constant*	0.1378*** (8.99)	0.1414*** (9.08)	0.1464*** (9.34)
*Industry*	YES	YES	YES
*Year*	YES	YES	YES
*N*	12,385	12,385	12,385
*Adj.R^2^*	0.269	0.269	0.270

Robust t–statistics in parentheses. ***p < 0.01, **p < 0.05, *p < 0.1.

For the firm's control variables, *Size, Firmage*, and *Roe* are negatively correlated with *CSR_rate* at a 1% significance level in column 3 of Table 6. Moreover, *Cash, Leverage*, and *Roa* are positively correlated with *CSR_rate* at a 1% significance level in column 3, which is in line with Cheung (2016). For the variables of ultimate controller trait, *Ucsgender* has a significant positive relationship with *CSR_rate*. In addition, *Ucsage* and *Ucsedu* are negatively correlated with *CSR_rate*. For regional characteristic variables, *GDP* is positively correlated with *CSR_rate*, while *Religion* is negatively correlated with *CSR_rate*. Therefore, our results hold H2, that is, when the place of incorporation is located in the expatriate hometown, HA of the ultimate controller who immigrated overseas is positively related to CSR.

### Further analysis

In this section, we explored the moderating effect of the personal characteristics of the ultimate controller and the characteristics of the region where the company is located on the relationship between HA and CSR.

#### Characteristics of the ultimate controller

Table 7 demonstrates the results of the moderating effect of ultimate controller heterogeneity on the relationship between HA and CSR. First, columns 1 and 2 of Table 7 report the moderating effect of gender of the ultimate controller. Specifically, in column 1, *HAhqhr_gender* is the cross-multiplier term of *HA_hqhr* and *Ucsgender*. It is shown that the estimated coefficient between *HAhqhr_gender* and *CSR_rate* is −0.0056 but statistically insignificant. It means that when the ultimate controller is male, the HA shows a negative relationship with CSR, but the result is statistically insignificant. Furthermore, in column 2, *HAqx_gender* is the cross-multiplier term of *HA_qx* and *Ucsgender*, and the estimated coefficient of *HAqx_gender* and *CSR_rate* is −0.0120 and significant at 10%. It is shown that male ultimate controllers in overseas Chinese hometown are significantly in a negative relationship with CSR. In other words, this result indicates that female entrepreneurs are more CSR-oriented, in line with the findings of Boulouta (2013). Therefore, the results generally support H3a, that is, female ultimate controllers positively moderate the relationship between HA and CSR.

**Table 7:** The moderating effect of characteristics of the ultimate controller (H3).

**Variables**	**(1)**	**(2)**	**(3)**	**(4)**	**(5)**	**(6)**
	***CSR_rate***	***CSR_rate***	***CSR_rate***	***CSR_rate***	***CSR_rate***	***CSR_rate***
*HA_hqhr*	0.0115** (2.05)		−0.0228** (−2.06)		0.0213*** (3.97)	
*HA_qx*		0.0218*** (3.70)		0.0068 (0.43)		0.0259*** (3.58)
*HAhqhr_gender*	−0.0056 (−0.96)					
*HAqx_gender*		−0.0120* (−1.85)				
*HAhqhr_age*			0.0550*** (2.65)			
*HAqx_age*				0.0075 (0.26)		
*HAhqhr_edu*					−0.0043*** (−3.13)	
*HAqx_edu*						−0.0044** (−2.32)
*Controls*	Control	Control	Control	Control	Control	Control
*Constant*	0.1458*** (9.27)	0.1454*** (9.28)	0.1502*** (9.48)	0.1467*** (9.33)	0.1459*** (9.28)	0.1468*** (9.37)
*Industry*	YES	YES	YES	YES	YES	YES
*Year*	YES	YES	YES	YES	Year	YES
*N*	12,385	12,385	12,385	12,385	12,385	12,385
*Adj. R^2^*	0.2730	0.2736	0.2734	0.2735	0.2736	0.2739

Robust t–statistics in parentheses. ***p < 0.01, **p < 0.05, *p < 0.1.

Next, columns 3 and 4 report the moderating effect of the age of the ultimate controller. Specifically, in column 3, *HAhqhr_age* is the cross-multiplier term of *HA_hqhr* and *Ucsage*, and the estimated coefficient of *HAhqhr_age* is 0.0550 and is significant at a 1% significance level. It implies that the age of the ultimate controller positively moderates the relationship between the ultimate controller with HA and CSR. Moreover, in column 4, *HAqx_age* is the cross-multiplier term of *HA_qx* and *Ucsage*, and the estimated coefficient of *HAhqhr_age* is 0.0075 but statistically insignificant. Therefore, the results generally support H3b, that is, as the age of the ultimate controller increases, the positive relationship between HA and CSR is greater.

Finally, columns 5 and 6 report that the education of the ultimate controller negatively moderates the relationship between HA and CSR. Specifically, in column 5, *HAhqhr_edu* is the cross-multiplier term of *HA_hqhr* and *Ucsedu*, and the estimated coefficient of *HAhqhr_edu* is −0.0043 and is significant at a 1% significance level. Additionally, in column 6, *HAqx_edu* is the cross-multiplier term of *HA_qx* and *Ucsedu*, and the estimated coefficient of *HAqx_edu* is −0.0044 and is significant at a 5% significance level. It is shown that the education of the ultimate controller negatively moderates the relationship between the ultimate controller with HA and CSR. Consistent with Hu et al. (2017), the results indicate that the ultimate controller with high education levels tends to be economically profitable. Therefore, the results support H3c, that is, the ultimate controller's education level negatively moderates the relationship between HA and CSR.

#### Marketization

Using the latest NERI index released by the National Economic Research Institute Reform Foundation (degree of marketization) (*Market*) as a benchmark (Fan et al., 2019), we grouped the sample by industry and annual median into a high marketization group (*Market_fast*) and a low marketization group (*Market_low*). We performed cross-sectional tests for model 1 and model 2, and Table 8 reports the results of the impact of marketization on the relationship between HA and CSR. Specifically, columns 1 and 2 present the correlation between *HA_hqhr* and *CSR_rate*. For the high marketization group in column 1, *HA_hqhr* is positively correlated with *CSR_rate* with an estimated coefficient of 0.0041, remaining statistically significant at the 10% significance level. Moreover, for the low marketization group in column 2, *HA_hqhr* is positively correlated with *CSR_rate* with an estimated coefficient of 0.0066, remaining statistically significant at the 5% significance level. Comparing columns 1 and 2, the coefficients of the *Market_low* group are 0.0025 higher than those of the *Market_high* group.

**Table 8:** The moderating effect of marketization (H4).

**Dependent variable**	**(1)**	**(2)**	**(3)**	**(4)**
	**Market_high**	**Market_low**	**Market_high**	**Market_low**
*HA_hqhr*	0.0041* (1.73)	0.0066** (2.44)		
*HA_qx*			0.0058* (1.93)	0.0132*** (2.95)
*Controls*	Control	Control	Control	Control
*Constant*	0.1598*** (6.60)	0.1400*** (6.59)	0.1596*** (6.60)	0.1393*** (6.55)
*Industry*	YES	YES	YES	YES
*Year*	YES	YES	YES	YES
*N*	5,953	6,429	5,953	6,429
*Adj. R^2^*	0.299	0.256	0.299	0.256

Robust t-statistics in parentheses. ***p < 0.01, **p < 0.05, *p < 0.1.

Next, columns 3 and 4 present the correlation between *HA_qx* and *CSR_rate*. Specifically, for the high marketization group in column 3, *HA_qx* is positively correlated with *CSR_rate* with an estimated coefficient of 0.0058, remaining statistically significant at the 10% significance level. In addition, for the low marketization group in column 4, *HA_qx* is positively correlated with *CSR_rate* with an estimated coefficient of 0.0132, remaining statistically significant at the 1% significance level. Comparing columns 3 and 4, the coefficients of the *Market_low* group are 0.0074 higher than those of the *Market_high* group.

These results support H4, that is, the degree of marketization negatively moderates the relationship between HA and CSR. Therefore, the results indicate that the positive influence of HA on CSR is greater in a low-market environment, and traditional culture has a greater influence on the behavioral decisions of overseas Chinese entrepreneurs.

#### Social trust

We used the China Commercial Credit Environment Index (CEI index) (*Trust*) as the benchmark and divide the trust level of the cities where the sample firms are registered into a higher trust level (*Trust_high*) and a lower trust level (*Trust_low*) according to the median. We conducted cross-sectional tests for model 1 and model 2, and Table 9 reports the effect of social trust on the relationship between HA and CSR. Specifically, columns 1 and 2 of Table 9 present the correlation between *HA_hqhr* and *CSR_rate*. For the high social trust group in column 1, *HA_hqhr* is positively correlated with *CSR_rate* with an estimated coefficient of 0.0065, remaining statistically significant at the 1% significance level. In addition, for the low social trust group in column 2, the estimated coefficient of *HA_hqhr* is 0.0038 but is statistically insignificant.

**Table 9:** The moderating effect of social trust (H5).

**Dependent variable**	**(1)**	**(2)**	**(3)**	**(4)**
	**Trust_high**	**Trust_low**	**Trust_high**	**Trust_low**
*HA_hqhr*	0.0065*** (2.96)	0.0038 (1.17)		
*HA_qx*			0.0124*** (4.24)	0.0078 (1.52)
*Controls*	Control	Control	Control	Control
*Constant*	0.1802*** (7.75)	0.1191*** (6.00)	0.1798*** (7.77)	0.1189*** (6.00)
*Industry*	YES	YES	YES	YES
*Year*	YES	YES	YES	YES
*N*	6,284	6,101	6,284	6,101
*Adj. R^2^*	0.261	0.314	0.262	0.314

Robust t-statistics in parentheses. ***p < 0.01.

Next, columns 3 and 4 of Table 9 present the correlation between *HA_qx* and *CSR_rate*. Specifically, for the high social trust group in column 3, *HA_qx* is positively correlated with *CSR_rate* with an estimated coefficient of 0.0124, remaining statistically significant at the 1% significance level. Additionally, for the low social trust group in column 4, the estimated coefficient of *HA_hqhr* is 0.0078 but is statistically insignificant.

The results support H5. Therefore, the results suggest that social trust positively moderates the relationship between HA and CSR among overseas Chinese entrepreneurs.

## Robustness tests

### Alternative measures of dependent and independent variables

We re-measured the independent variable HA (*HA_qx*). Since 2014, China has set up national-level overseas Chinese industrial zones and entrepreneurial clusters – QiaoMengYuan (QMY), i.e., Overseas Chinese Dream Zone – to attract overseas Chinese businessmen and talents to invest and start businesses. As of 2019, China has established seventeen QMYs across the country. The areas where the QMYs have been established have attracted more overseas businessmen to gather. Areas, where locals and overseas Chinese firms have enhanced ties, are more likely to be close to the traditional diaspora atmosphere. Thus, we used *HA_qmy* as a proxy for the independent variable *HA_qx*. We defined *HA_qmy* as a dummy variable that equals 1 if the firm's place of incorporation is in the province where QMY is located and 0 otherwise. Table 10 shows that the estimated coefficient of *HA_qmy* is 0.0131 and is significantly positive at the 1% level of significance. Therefore, our results remain robust.

**Table 10:** Changing key variables.

**Variables**	**(1)**
	***CSR_rate***
*HA_qmy*	0.0131*** (5.50)
*Controls*	Control
*Constant*	0.1461*** (9.34)
*Industry*	YES
*Year*	YES
*N*	12,385
*Adj. R^2^*	0.271

Robust t-statistics in parentheses. ***p < 0.01.

### Exclude the impact of the financial crisis

Overseas-funded enterprises invested by overseas Chinese entrepreneurs are more vulnerable to the overseas market environment. Considering that the financial crisis has a certain impact on their social responsibility performance, we excluded the impact of the 2008 financial crisis and selected the regressions for the sample of firms from 2009 to 2019. Table 11 reports the regression results. Specifically, in column 1, *HA_hqhr* is positively correlated with *CSR_rate* with an estimated coefficient of 0.0055, remaining statistically significant at the 1% significance level. In addition, in column 2, *HA_qx* is positively correlated with *CSR_rate* with an estimated coefficient of 0.0095, remaining statistically significant at the 1% significance level. It is shown that HA of the ultimate controller promotes CSR. Therefore, our results are robust.

**Table 11:** Excluding the impact of the financial crisis.

**Dependent variable**	**(1)**	**(2)**
	**Nocrisis**	**Nocrisis**
*HA_hqhr*	0.0055*** (2.85)	
*HA_qx*		0.0095*** (3.50)
*Controls*	Control	Control
*Constant*	0.1900*** (11.43)	0.1893*** (11.41)
*Industry*	YES	YES
*Year*	YES	YES
*N*	11,065	11,065
*Adj. R^2^*	0.268	0.268

Robust t-statistics in parentheses. ***p < 0.01.

### Endogeneity

Although we have controlled for the effects of economic and different cultural factors, there may still be omitted variables. Following Ang et al. (2014), we used the instrumental variables approach (2SLS) to address possible endogeneity issues. Specifically, we selected two instrumental variables. One is the number of coastal ports of commerce that China was forced to open under unequal treaties between 1840 and the end of the 19th century (*Tradingport*). The other is a dummy variable that equals 1 if Christian and Roman Catholic missionaries opened Western-style colleges (*Westcollege*) in a region of China as of the 1820s and 0 otherwise.

The above two instrumental variables are theoretically exogenous and relevant. First, from the exogeneity perspective, these two variables can meet the exogeneity requirement better because their data sources are old and not directly related to contemporary CSR behaviors. Second, in terms of relevance, Zhuang (2011) has found that the large-scale Chinese workers' emigration in modern history was caused by the surge of labor demand due to the warfare starting from the Second Opium War and the development of trade in the colonies belonging to Europe and America, and the opening of coastal ports of commerce accelerated the process of Chinese workers' emigration. In addition, people in the areas where western-style universities were founded received western ideas earlier and their likelihood to develop or migrate abroad increased when faced with the chaotic situation and poor survival conditions at home after the Opium War. Therefore, these two instrumental variables can better satisfy the requirement of correlation.

Next, we statistically validated the exogeneity and correlation of the instrumental variable to address the endogeneity of the ultimate controller's HA and CSR. Table 12 shows the 2SLS regression results for the instrumental variables. Column 1 reports the results of the first-stage regression and the estimated coefficients of *Tradingport* and *Westcollge* are significantly positive at the 1% significance level. The results indicate that the ultimate controller of the local firm is more likely to be an overseas Chinese if the province where the firm is located has a higher number of open ports of commerce and operates a Western-style university, and the result is consistent with our expectations. Column 2 presents the results of the second-stage regression, where the estimated coefficient of the independent variable *HA_hqhr* is significantly positive at the 1% significance level, consistent with the results of the baseline regression.

**Table 12:** Instrumental variables regression.

**Variables**	**(1)**	**(2)**
	**First stage**	**Second stage**
	**HA_hqhr**	**CSR_rate**
*Tradingport*	0.0106*** (5.75)	
*Westcollge*	0.0277*** (3.16)	
*HA_hqhr*		0.0562*** (2.75)
*Control variables*	Control	Control
*Intercept*	0.3287*** (3.71)	0.1276*** (7.17)
*Industry*	YES	YES
*Year*	YES	YES
*N*	12,385	12,385
*Adj. R^2^*	0.0558	0.2228
Kleibergen-Paap rk LM statistic: 89.2880, *P* = 0.0000		
Kleibergen-Paap rk Wald F statistic: 45.1720 [19.93]		
Hansen J statistic: 0.6870, *P* = 0.4070		
Endogeneity test: 6.1410, *P* = 0.0132		

*** represents significance levels at the 1% levels. In column 1, the t-statistics in parentheses are clustered from the robust standard errors clustered at the firm level. In column 2, the z-statistics in parentheses are clustered from the robust standard errors clustered at the firm level. Kleibergen-Paap rk LM statistic represents the result of the “unidentifiable” test, and the *p*-value = 0.0000 rejects the null hypothesis of “unidentifiable”. The Kleibergen-Paap rk Wald F statistic represents the results of the “weak instrumental variable” test, and the 10% threshold of the Stock-Yogo weak instrumental variable test is in parentheses. The Hansen J statistic represents the results of the “overidentification” test, with the null hypothesis that “all instrumental variables are exogenous”. The endogeneity test is the result of the endogeneity test of the independent variable, and the null hypothesis is “the independent variable is exogenous”.

Finally, in the instrumental variable test, we found that the Kleibergen-Paap rk Wald F-statistic is greater than the 10% threshold of the corresponding Stock-Yogo weak instrumental variable test, which indicates that the null hypothesis of “the existence of weak instrumental variables” can be significantly rejected, i.e., the instrumental variables *Tradingport* and *Westcollge* satisfy the requirement of correlation. The *p*-value of the Hansen J statistic is 0.4070, which indicates that the null hypothesis of “all instrumental variables are exogenous” cannot be rejected, i.e., the instrumental variables *Tradingport* and *Westcollge* satisfy the requirement of exogeneity. In addition, the regression results also reported the endogeneity test of the independent variable *HA_hqhr* with a *p*-value of 0.0132, indicating that the null hypothesis of “the independent variable is exogenous” can be rejected and that the HA variable has some endogeneity problems, so it is necessary to use the instrumental variables method to solve the endogeneity problem. In conclusion, after controlling for the endogeneity problem caused by omitted variables using the instrumental variables method, our results are still robust.

### Summary

With the above analysis, we summarized the results of our hypotheses tests in Table 13. In detail, we examined the effect of HA on CSR by baseline regression, and the results verified that HA promotes CSR, i.e., H1 and H2. Next, we examined the moderating effect of ultimate controller characteristics (i.e., gender, age, and education) on the HA-CSR relationship. The regression results validate that the female ultimate controller positively moderates the HA-CSR relationship, i.e., H3a, that age of the ultimate controller positively moderates the HA-CSR relationship, i.e., H3b, and that the education level of the ultimate controller negatively moderates the HA-CSR relationship, i.e., H3c. Furthermore, we tested the moderating effect of regional characteristics (i.e., marketization and social trust) on the HA-CSR relationship. The results reveal that marketization negatively regulates the HA-CSR relationship, i.e., H4, and social trust positively regulates the HA-CSR relationship, i.e., H5. Overall, our hypotheses have been validated.

**Table 13:** Results of the hypotheses.

**Hypothesis**	**Mechanism**	**Regression test**	**Result**
H1	HA → CSR	+	Accepted
H2	HA → CSR	+	Accepted
H3a	Gender × HA → CSR	-	Accepted
H3b	Age × HA → CSR	+	Accepted
H3c	Education × HA → CSR	-	Accepted
H4	Marketization × HA → CSR	-	Accepted
H5	Social Trust × HA → CSR	+	Accepted

## Conclusions and discussions

### Discussions

To explore the effect of HA of the firm's ultimate controller on CSR, this study combines identity theory, attachment theory, and upper echelons theory to empirically examine the relationship between the HA of the ultimate controller of firms with overseas migration experience and CSR using a sample of Chinese non-financial private-listed firms from 2003 to 2019.

The results show that H1 and H2 are supported. The HA of the ultimate controller has a positive impact on CSR in Chinese-listed private firms. This result is consistent with the results of Ren et al. (2021a), who studied the relationship between CEO hometown identity and firm innovation. It is worth noting that firm innovation is a sustainable firm behavior that contributes to the long-term growth of the firm. In other words, hometown identity of firm managers and attachment motivate them to implement what is good for their hometown and firm. Our study finds that the HA of the ultimate controllers motivates firms to fulfill their social responsibility. In addition, there are some studies that suggest that CEOs use hometown ties to avoid taxes and gain more financial benefits for their companies. For example, Shen et al. (2019) argued that CEOs' hometown ties with local government officials motivate firms to avoid taxes. Clearly, tax avoidance is a behavior that only benefits the firm but is detrimental to fulfilling social responsibility. Unlike Shen et al. (2019), this study examines the HA of the ultimate controllers rather than the CEOs' hometown ties. It is shown that attachment triggers managers to do good things for their hometown, while hometown ties may prompt managers to be more inclined to seek financial benefits. In addition, we chose the perspective of ultimate controllers who have overseas experience as our study subjects. Considering the decisive role that ultimate controllers have on corporate decision-making, we selected the HA of ultimate controllers as our research.

The results support H3a–H3c. Female ultimate controllers positively moderate the relationship between HA and CSR in Chinese-listed private firms. Our findings are consistent with Boulouta (2013) who found that female managers are more concerned with social responsibility. In contrast to the above findings, this study examines the role female managers play in CSR based on the psychology of HA. Our descriptive statistics find a larger proportion of male ultimate controllers and a smaller proportion of female ultimate controllers in our sample observations, in line with Kong et al. (2020). Although this result indicates that female entrepreneurs still account for a smaller share of management, it is clear from our regression results that female final controllers play a positive influence on CSR concerns and decisions, and that stronger attachment of female is the important mechanism. Furthermore, our results discover that age positively moderates the effect of ultimate controller HA on CSR. This result differs from the results of Hu et al. (2017), who examined the effect of hometown identification on corporate environmental governance, while we examined the effect of HA on CSR. Notably, environmental governance is part of CSR and younger ultimate controllers may have more environmental education and be more environmentally conscious, thus as Hu et al. (2017) found that the role of hometown identification of younger chairmen on corporate environmental investment is greater. However, CSR is an integrated behavior that includes behaviors such as the welfare of employees and philanthropic activities besides environmental behaviors. In addition, the education level of the ultimate controller negatively moderates the effect of HA on CSR, consistent with Hu et al. (2017).

The results support H4. Among Chinese-listed private firms, the extent of marketization negatively moderates the relationship between the HA of the ultimate controller and CSR. Our results show that the HA of the ultimate controller has less impact on CSR in regions with a higher degree of marketization. Thus, as the degree of marketization increases, firms' decisions become more rational and may be less influenced by traditional culture.

The results support H5. Social trust positively moderates the relationship between ultimate controllers' HA and CSR in Chinese-listed private firms. Our results show that HA of ultimate controllers has a stronger incentive effect on CSR in regions with high levels of social trust. Notably, social trust is an important condition for economic activities, and our study adds to evidence of the impact of social trust on non-economic activities of firms, i.e., CSR.

### Conclusion

We empirically tested the relationship between the HA of the firm's ultimate controller and CSR using a sample of Chinese non-financial private listed firms from 2003 to 2019. We proxied the HA of the ultimate controller by the Chinese diaspora identity of the ultimate controller, and the results showed that HA helps to improve the level of CSR. Moreover, the level of CSR is higher when the ultimate controller is an overseas immigrant and the company is registered in the expatriate hometown. Furthermore, we examined the moderating effect of the characteristics of the ultimate controller on HA and CSR. Our results found that among listed private firms, female ultimate controller and ultimate controller age positively moderate the relationship between HA and CSR, while ultimate controller education level negatively moderates the relationship. In addition, we examined the moderating effect of the external environment on the relationship between HA and CSR. The results show that the level of social trust in the place of incorporation of listed companies positively moderates the relationship between HA and CSR, while the degree of marketization in the place of incorporation of listed companies negatively moderates the relationship. Finally, our results still hold after the endogeneity test and robustness test.

This paper has the following three implications for existing theory and management practice. First, we have enriched the research on the motivation of CSR. Although there has been much literature examining the economic motivations of CSR, there are still fewer studies that explore CSR motivations from a psychological perspective. For example, Ren et al. (2021a) explored the influence of hometown identity on CEO decision-making, but we studied HA from the perspective of overseas immigrants and developed the influence of attachment psychology on CSR.

Second, we have enriched the research related to attachment psychology and identity theory. Existing studies have mainly explored the well-being experiences or pro-social behaviors resulting from place attachment (e.g., Vada et al., 2019; Daryanto and Song, 2021). However, the possible effects of attachment on firms have been understudied. In business management, HA is a key factor that cannot be ignored. Not only do managers' personal experiences as well as their personalities influence decision-making, but employees' psychology also affects the daily operations of the company. Therefore, it is necessary for managers to pay attention to the economic consequences of attachment psychology.

Third, we have advanced interdisciplinary research. Previous research on migration has mostly focused on psychological and sociological perspectives, and research on the impact on the business domain remains scarce. For example, Dai et al. (2011) and Mata and Alves (2018) explored immigrants' adaptation from their entrepreneurship in the host country, but we discussed the psychology of immigrant entrepreneurship from a different perspective, i.e., back home country.

This paper has the following limitations and perspectives. First, this paper only examines attachment psychology that is only one of the psychological factors of overseas immigrants. Therefore, future literature can explore the impact of other psychological factors of immigrants on CSR. Second, this paper only explores immigrants' investment and entrepreneurship in their home countries to improve CSR. Future studies can examine the relationship between immigrants' entrepreneurship and CSR in the host country and its impact mechanism. Third, the characteristics and experiences of the ultimate controller as a decision-maker have an important impact on corporate strategy. The impact of the attachment psychology of the ultimate controller on other aspects of the firm deserves further investigation.

This study has the following policy implications. In the context of economic globalization and growing migration, the role played by managers with expatriate experience in corporate decision-making has received increasing attention. Notably, attachment psychology is pervasive among immigrants and potentially influences their decision-making and behavior. Out of attachment psychology, immigrants tend to establish ties with their hometown and do good deeds for them. Accordingly, managers with immigrant experiences are more concerned about CSR. Therefore, the government should provide policy support to encourage entrepreneurs with overseas immigrant backgrounds to return to their home countries so as to increase their willingness to invest, fully utilize foreign capital and advanced overseas business operation concepts, and facilitate the fulfillment of CSR.

## Data availability statement

The raw data supporting the conclusions of this article will be made available by the authors, without undue reservation.

## Author contributions

JX: conceptualization, data curation, formal analysis, investigation, software, and writing—original draft. ZW: conceptualization, funding acquisition, investigation, supervision, and writing—review and editing. ZD: conceptualization, data curation, investigation, validation, and writing—review and editing. RZ: writing—review and editing, validation, and methodology. All authors contributed to the article and approved the submitted version.

## Funding

This study is financially supported by the National Natural Science Foundation of China (Grant Nos.: 72173057, 7211101263, and 71672077), Natural Science Foundation of Guangdong Province, China (2021A1515011536), and Fundamental Research Funds for the Central Universities (19JNKY08).

## Conflict of interest

The authors declare that the research was conducted in the absence of any commercial or financial relationships that could be construed as a potential conflict of interest.

## Publisher's note

All claims expressed in this article are solely those of the authors and do not necessarily represent those of their affiliated organizations, or those of the publisher, the editors and the reviewers. Any product that may be evaluated in this article, or claim that may be made by its manufacturer, is not guaranteed or endorsed by the publisher.

## Footnotes

^1^The address of the website of *cninf* : http://www.cninfo.com.cn.

^2^The website address of All-China Federation of Returned Overseas Chinese is http://www.chinaql.org. In addition, we also searched for provincial federations of returned overseas Chinese, such as Guangdong Province Federation of Returned Overseas Chinese: http://www.gdql.org.cn; Zhejiang Province Federation of Returned Overseas Chinese: http://www.zjsql.com.cn; etc.

^3^Based on the distribution of sample companies by industry (Table 1), and descriptive statistics of companies' financial data (Table 3), it appears that our selected sample is not significantly different from the data of previous literature studying private companies, such as Chen et al. (2020), Kong et al. (2020), and Ren et al. (2021a). Furthermore, the results based on the year distribution statistics of the ultimate controllers (Table 2) and the univariate test (Table 4) show that there is a significant difference between the CSR of the experimental and control groups, laying the foundation for our regression analysis.

^4^The website address of National Bureau of Statistics is http://www.stats.gov.cn.

^5^The website address of National Religious Affairs Administration is https://www.sara.gov.cn/gjzjswjhtml/index.html. In addition, the list of award winners is the religious places that won the “Third Session of Advanced Collective and Advanced Individuals for Creating Harmonious Monasteries and Churches” in 2016, and the relevant data have been compiled manually by the author.

^6^The website addresses of the provincial Federations of Industry and Commerce include Beijing Federation of Industry and Commerce: http://www.bjgsl.org.cn; Guangdong Federation of Industry and Commerce: http://www.gdgcc.gov.cn; Zhejiang Federation of Industry and Commerce: http://www.zjfic.org.cn; etc.

## References

[B1] Adger W. N. Barnett J. Brown K. Marshall N. 'Brien K. (2013). Cultural dimensions of climate change impacts and adaptation. Nat. Clim. Chang. 3, 112–117. 10.1038/nclimate166631385093

[B2] Ahmed R. R. Streimikiene D. (2021). Environmental issues and strategic corporate social responsibility for organizational competitiveness. J. Compet. 13, 5–22. 10.7441/joc.2021.02.01

[B3] Aktas N. De Bodt E. Bollaert H. Roll R. (2016). CEO narcissism and the takeover process: From private initiation to deal completion. J. Financ. Quant. Anal. 51, 113–137. 10.1017/S0022109016000065

[B4] Allen F. Qian J. Qian M. (2005). Law, finance, and economic growth in China. J. Financ. Econ. 77, 57–116. 10.1016/j.jfineco.2004.06.010

[B5] Ang J. S. Cheng Y. Wu C. (2014). Does enforcement of intellectual property rights matter in China? Evidence from financing and investment choices in the high-tech industry. Rev. Econ. Stat. 96, 332–348. 10.1162/REST_a_00372

[B6] Anton C. E. Lawrence C. (2014). Home is where the heart is: The effect of place of residence on place attachment and community participation. J. Environ. Psychol. 40, 451–461. 10.1016/j.jenvp.2014.10.007

[B7] Berg J. Dickhaut J. McCabe K. (1995). Trust, reciprocity, and social history. Games Econ. Behav. 10, 122–142. 10.1006/game.1995.1027

[B8] Berry J. W. Phinney J. S. Sam D. L. Vedder P. (2006). Immigrant youth: Acculturation, identity, and adaptation. Appl. Psychol.-Int. Rev. 55, 303–332. 10.1111/j.1464-0597.2006.00256.x

[B9] Boulouta I. (2013). Hidden connections: The link between board gender diversity and corporate social performance. J. Bus. Ethics 113, 185–197. 10.1007/s10551-012-1293-7

[B10] Brown K. Adger W. N. Devine-Wright P. Anderies J. M. Barr S. Bousquet F.. (2019). Empathy, place and identity interactions for sustainability. Glob. Environ. Change-Human Policy Dimens. 56, 11–17. 10.1016/j.gloenvcha.2019.03.003

[B11] Burke S. Zvarikova K. (2021). Urban Internet of Things systems and data monitoring algorithms in smart and environmentally sustainable cities. Geopolit. Hist. Int. Relat. 13, 135–148. 10.22381/GHIR132202110

[B12] Chen C. C. Chen Y.-R. Xin K. (2004). Guanxi practices and trust in management: A procedural justice perspective. Organ. Sci. 15, 200–209. 10.1287/orsc.1030.0047

[B13] Chen T. Dong H. Lin C. (2020). Institutional shareholders and corporate social responsibility. J. Financ. Econ. 135, 483–504. 10.1016/j.jfineco.2019.06.007

[B14] Cheung A. (2016). Corporate social responsibility and corporate cash holdings. J. Corp. Financ. 37, 412–430. 10.1016/j.jcorpfin.2016.01.00834580557

[B15] Coval J. D. Moskowitz T. J. (1999). Home bias at home: Local equity preference in domestic portfolios. J. Financ. 54, 2045–2073. 10.1111/0022-1082.00181

[B16] Crane A. Matten D. (2020). COVID-19 and the future of CSR research. J. Manage. Stud. 58, 280–284. 10.1111/joms.12642

[B17] Crisan-Mitra C. S. Stanca L. Dabija D.-C. (2020). Corporate social performance: An assessment model on an emerging market. Sustainability 12, 4077. 10.3390/su12104077

[B18] Cronqvist H. Yu F. (2017). Shaped by their daughters: Executives, female socialization, and corporate social responsibility. J. Financ. Econ. 126, 543–562. 10.1016/j.jfineco.2017.09.003

[B19] Dai F. Wang K. Y. Teo S. T. (2011). Chinese immigrants in network marketing business in Western host country context. Int. Bus. Rev. 20, 659–669. 10.1016/j.ibusrev.2011.02.015

[B20] Dai Y. F. Song P. (1998). Review and prospects of Fujian Overseas Chinese studies. J. Overseas Chin. Hist. Stud. 1, 38–47.

[B21] Daryanto A. Song Z. (2021). A meta-analysis of the relationship between place attachment and pro-environmental behaviour. J. Bus. Res. 123, 208–219. 10.1016/j.jbusres.2020.09.045

[B22] Davidson R. H. Dey A. Smith A. J. (2019). CEO materialism and corporate social responsibility. Account. Rev. 94, 101–126. 10.2308/accr-52079

[B23] Dubno P. (1985). Attitudes toward women executives: A longitudinal approach. Acad. Manage. J. 28, 235–239. 10.5465/256072

[B24] Fan G. Ma G. Wang X. (2019). Institutional reform and economic growth of China: 40-year progress toward marketization. Acta. Oecon. 69, 7–20. 10.1556/032.2019.69.s1.2

[B25] Gifford R. (2014). Environmental psychology matters. Annu. Rev. Psychol. 65, 541–579. 10.1146/annurev-psych-010213-115048 24050189

[B26] Hambrick D. C. Mason P. A. (1984). Upper echelons: The organization as a reflection of its top managers. Acad. Manage. Rev. 9, 193–206. 10.2307/258434

[B27] Harris L. J. (2015). Overseas Chinese remittance firms, the limits of state sovereignty, and transnational capitalism in East and Southeast Asia, 1850s-1930s. J. Asian Stud. 74, 129–151. 10.1017/S0021911814001697

[B28] Hodler R. Raschky P. A. (2014). Regional favoritism. Q. J. Econ. 129, 995–1033. 10.1093/qje/qju004

[B29] Hu J. Song X. Wang H. (2017). Informal institution, hometown identity and corporate environmental governance. Manag. World 3, 76–94. 10.19744/j.cnki.11-1235/f.2017.03.006

[B30] Hu X. M. Wang Y. Liao S. H. Peng K. P. (2021). Do experiences studying abroad promote dialectical thinking? Empirical evidence from chinese international students. Front. Psychol. 12, 595935. 10.3389/fpsyg.2021.595935 34122210 PMC8195590

[B31] Huang W.-J. Hung K. Chen C.-C. (2018). Attachment to the home country or hometown? Examining diaspora tourism across migrant generations. Tour. Manage. 68, 52–65. 10.1016/j.tourman.2018.02.019

[B32] King R. Christou A. (2014). Second-generation “Return” to Greece: New dynamics of transnationalism and integration. Int. Migr. 52, 85–99. 10.1111/imig.12149

[B33] Kong D. Pan Y. Tian G. G. Zhang P. (2020). CEOs' hometown connections and access to trade credit: Evidence from China. J. Corp. Financ. 62, 101574. 10.1016/j.jcorpfin.2020.101574

[B34] Kong F. Z. Zhao L. Zhang X. B. Tsai C. H. Lin D. D. (2019). Farmers' work-life quality and entrepreneurship will in China. Front. Psychol. 10, 787. 10.3389/fpsyg.2019.00787 31114517 PMC6502897

[B35] Lee T. H. (2011). How recreation involvement, place attachment and conservation commitment affect environmentally responsible behavior. J. Sustain. Tour. 19, 895–915. 10.1080/09669582.2011.570345

[B36] Ma L. J. Xiang B. (1998). Native place, migration and the emergence of peasant enclaves in Beijing. China Q. 155, 546–581. 10.1017/S0305741000049997

[B37] Masterson V. A. Stedman R. C. Enqvist J. Tengo M. Giusti M. Wahl D.. (2017). The contribution of sense of place to social-ecological systems research: a review and research agenda. Ecol. Soc. 22, 49. 10.5751/ES-08872-220149

[B38] Masulis R. W. Reza S. W. (2015). Agency problems of corporate philanthropy. Rev. Financ. Stud. 28, 592–636. 10.1093/rfs/hhu082

[B39] Mata J. Alves C. (2018). The survival of firms founded by immigrants: Institutional distance between home and host country, and experience in the host country. Strateg. Manage. J. 39, 2965–2991. 10.1002/smj.2945

[B40] May A. Y. C. Hao G. S. Carter S. (2021). Intertwining corporate social responsibility, employee green behavior, and environmental sustainability: The mediation effect of organizational trust and organizational identity. Econ. Manag. Financ. Mark. 16, 32–61. 10.22381/emfm16220212

[B41] Michel J. G. Hambrick D. C. (1992). Diversification posture and top management team characteristics. Acad. Manage. J. 35, 9–37. 10.5465/256471

[B42] Narver J. C. Slater S. F. (1990). The effect of a market orientation on business profitability. J. Mark. 54, 20–35. 10.1177/0022242990054004039776938

[B43] Norburn D. Birley S. (1988). The top management team and corporate performance. Strateg. Manage. J. 9, 225–237. 10.1002/smj.4250090303

[B44] Palladino S. (2019). Older migrants reflecting on aging through attachment to and identification with places. J. Aging Stud. 50, 100788. 10.1016/j.jaging.2019.100788 31526495

[B45] Petrenko O. V. Aime F. Ridge J. Hill A. (2016). Corporate social responsibility or CEO narcissism? CSR motivations and organizational performance. Strateg. Manage. J. 37, 262–279. 10.1002/smj.2348

[B46] Ren S. Cheng Y. Hu Y. Yin C. (2021a). Feeling right at home: Hometown CEOs and firm innovation. J. Corp. Financ. 66, 101815. 10.1016/j.jcorpfin.2020.101815

[B47] Ren S. Wang Y. Hu Y. Yan J. (2021b). CEO hometown identity and firm green innovation. Bus. Strateg. Environ. 30, 756–774. 10.1002/bse.2652

[B48] Scannell L. Gifford R. (2013). Personally relevant climate change: The role of place attachment and local versus global message framing in engagement. Environ. Behav. 45, 60–85. 10.1177/0013916511421196

[B49] Shen Y. Gao D. Bu D. Yan L. Chen P. (2019). CEO hometown ties and tax avoidance-evidence from China's listed firms. Account. Financ. 58, 1549–1580. 10.1111/acfi.12442

[B50] Song J. B. Li A. H. (2010). Research on corporate governance factors of corporate social responsibility Res. Financ. Econ. Issues 318, 23–29. 10.19654/j.cnki.cjwtyj.2010.05.004

[B51] Tetrault Sirsly C.-A. Lvina E. (2019). From doing good to looking even better: The dynamics of CSR and reputation. Bus. Soc. 58, 1234–1266. 10.1177/0007650315627996

[B52] Trevino L. K. (1986). Ethical decision making in organizations: A person-situation interactionist model. Acad. Manage. Rev. 11, 601–617. 10.2307/258313

[B53] Udayasankar K. (2008). Corporate social responsibility and firm size. J. Bus. Ethics 83, 167–175. 10.1007/s10551-007-9609-835035363

[B54] Vada S. Prentice C. Hsiao A. (2019). The influence of tourism experience and well-being on place attachment. J. Retail. Consum. Serv. 47, 322–330. 10.1016/j.jretconser.2018.12.00735865700

[B55] Wang Y. L. (2001). The characteristics of the development of the relations between overseas Chinese and their hometowns. J. Jinan Univ. Philos. Soc. Sci. 23, 129–134. Available online at: https://kns.cnki.net/kcms/detail/detail.aspx? dbcode=CJFD&dbname=CJFD2001&filename=JNXB200104021&v=MjUxMDBl WDFMdXhZUzdEaDFUM3FUcldNMUZyQ1VSN2lmYnVkc0ZDamtWTHpMT HlQVGJMRzRIdERNcTQ5SFpZUjg=

[B56] Wang Z. Guo M. Ming J. (2020). Effect of hometown housing investment on the homeownership of rural migrants in urban destinations: Evidence from China. Cities 105, 102619. 10.1016/j.cities.2020.102619

[B57] Watanabe M. (2002). Holding company risk in China: A final step of state-owned enterprises reform and an emerging problem of corporate governance. China Econ. Rev. 13, 373–381. 10.1016/S1043-951X(02)00094-9

[B58] Wessendorf S. (2010). Local attachments and transnational everyday lives: second-generation Italians in Switzerland. Glob. Netw. 10, 365–382. 10.1111/j.1471-0374.2010.00293.x

[B59] Wiersema M. F. Bantel K. A. (1992). Top management team demography and corporate strategic change. Acad. Manage. J. 35, 91–121. 10.5465/256474

[B60] Zaušková A. Rezníčková M. (2020). SoLoMo marketing as a global tool for enhancing awareness of eco-innovations in Slovak business environment. Equilib. Q. J. Econ. Econ. Policy 15, 133–150. 10.24136/eq.2020.007

[B61] Zhang J. Kong D. Wu J. (2018). Doing good business by hiring directors with foreign experience. J. Bus. Ethics 153, 859–876. 10.1007/s10551-016-3416-z

[B62] Zheng D. H. (2009). A re-examination of the concept of “diaspora” and its research. Acad. Res. 2, 95–100. Available online at: https:// kns.cnki.net/kcms/detail/detail.aspx?dbcode=CJF&dbname=CJFD2009&filename =XSYJ200902019&v=MTk3ODJDVVI3aWZidWRzRkNqbFZidklQVDdTWkxHN Eh0ak1yWTlFYllSOGVYMUx1eFlTN0RoMVQzcVRyV00xRnI=20706783

[B63] Zhou M. (2009). How neighbourhoods matter for immigrant children: The formation of educational resources in Chinatown, Koreatown and Pico Union, Los Angeles. J. Ethn. Migr. Stud. 35, 1153–1179. 10.1080/13691830903006168

[B64] Zhuang G. T. (2011). Historical changes in numbers and distribution of overseas Chinese in the world. World Hist. 2011, 4–14, 157. Available online at: https://kns.cnki.net/kcms/detail/detail.aspx?dbcode=CJFD&dbname=CJFD2011& filename=HIST201105003&v=MTY4MDFTVFllckc0SDlETXFvOUZaNFI4ZVgxT HV4WVM3RGgxVDNxVHJXTTFGckNVUjdpZmJ1ZHNGQ2ptVmIzSUw=

[B65] Zingales L. (2015). The “cultural revolution” in finance. J. Financ. Econ. 117, 1–4. 10.1016/j.jfineco.2015.05.006

[B66] Zou Y. Meng F. Li Q. (2021). Chinese diaspora tourists' emotional experiences and ancestral hometown attachment. Tour. Manag. Perspect. 37, 100768. 10.1016/j.tmp.2020.100768

